# Generative artificial intelligence is infiltrating peer review process

**DOI:** 10.1186/s13054-024-04933-z

**Published:** 2024-05-07

**Authors:** Kunming Cheng, Zaijie Sun, Xiaojun Liu, Haiyang Wu, Cheng Li

**Affiliations:** 1 Department of Intensive Care Unit, The Second Affiliated Hospital of Zhengzhou University, Zhengzhou, China; 2 Department of Orthopaedics, Xiangyang Central Hospital, Affiliated Hospital of Hubei University of Arts and Science, Xiangyang, China; 3 Department of Orthopaedics, The First Affiliated Hospital of Zhengzhou University, Zhengzhou, China; 4 Department of Clinical College of Neurology, Neurosurgery and Neurorehabilitation, Tianjin Medical University, Tianjin, China; 5 Department of Orthopaedic Surgery, Beijing Jishuitan Hospital, Capital Medical University, Beijing, China; 6 Center for Musculoskeletal Surgery (CMSC), Charité-Universitätsmedizin Berlin, Corporate Member of Freie Universität Berlin, Berlin Institute of Health, Humboldt University of Berlin, Berlin, Germany

The advancement of scientific research has been rapid in recent years, leading to a surge in the number of manuscript submissions and posing formidable challenges to peer review processes. In addressing these challenges, some generative artificial intelligence (AI) tools have emerged as potentially effective solutions [1, 2]. For instance, Saad et al. [3] explored the efficiency and efficacy of one such generative AI tool, the ChatGPT, in the peer review process. Each article underwent review by two human reviewers alongside ChatGPT 3.5 and ChatGPT 4. ChatGPT was tasked with providing three positive and three negative comments on the articles, along with recommendations for acceptance or rejection. Their findings demonstrated ChatGPT was able to complement human scientific peer review, improving the efficiency and timeliness of the editorial process. Verharen et al. [4] utilized ChatGPT to examine language usage in over 500 publicly available peer review reports from 200 neuroscience papers published between 2022 and 2023. The findings revealed that the majority of reviews for these published papers were deemed favorable by ChatGPT (89.8% of reviews), with language use characterized as predominantly polite (99.8% of reviews). This study underscores the potential of generative AI in natural language processing of specialized scientific texts. However, careful consideration is warranted in balancing the roles of AI tools and human experts to ensure fairness and reliability in the peer review process.

One recent study has compared the use of adjectives in over 146,000 peer reviews submitted to the same conference before and after the advent of ChatGPT [5]. Analysis revealed a significant increase in the frequency of certain positive adjectives, such as commendable, innovative, notable and versatile, since the integration of the chatbot into the mainstream. However, some scholars speculate that this phenomenon may stem from non-native English-speaking reviewers using ChatGPT for adjusting and refining English writing. Given the gradual infiltration of generative AI tools into academic peer review, scholarly publishers and relevant institutions have begun issuing regulations regarding the use of such tools in the peer review process.

On June 23, 2023, the National Institutes of Health (NIH) implemented a ban on the use of online generative AI tools like ChatGPT for analysis and drafting of peer review comments. The Australian Research Council (ARC) also prohibited the use of generative AI in peer review. Concerning journals, the latest recommendations from the International Committee of Medical Journal Editors (ICMJE) suggest that reviewers should not upload manuscripts to software or other AI technology platforms that cannot guarantee confidentiality. Reviewers should disclose to the journal whether and how AI technology was used in evaluating manuscripts or drafting reviewer comments. The journal *Science* prohibits the use of large language models during peer review and prohibits reviewers from uploading manuscripts to generative AI tools. The *Lancet* maintains that reviewers should refrain from using generative AI or AI-assisted technologies to assist in the scientific review of papers. Reviewers must treat papers shared by editors as confidential during the peer review process and should not upload papers or any part thereof to AI tools. This is because the critical thinking and assessment of research originality required in peer review extend beyond the scope of this technology, posing certain risks such as generating incorrect, incomplete, or biased conclusions about manuscript submissions. In addition, JAMA now includes in its reviewer instructions the following: entering any portion of the manuscript, abstract, or reviewer comments into chatbots, language models, or similar tools violates their confidentiality agreement. If the reviewers use an AI tool in a manner that does not violate the journal’s confidentiality policy, they must provide the name and usage method of the tool.

We also further summarized the requirements for the peer review process of the top ten journals with the highest impact factor in the field of critical care medicine. As shown in Table 1, with the exception of *Lancet Respiratory Medicine*, the other nine journals have no statement on AI and AI-assisted technologies in peer review process. Therefore, we call on relevant journals to take action and promptly update their policies on the use of AI tools in peer review.

**Table 1:** Statement on AI and AI-assisted technologies in peer review in the top ten journals with the highest impact factor in the field of critical care medicine

Journals	Statement on AI and AI-assisted technologies in peer review
Lancet Respiratory Medicine	1. Reviewers should not use artificial intelligence (AI) or AI-assisted technologies to assist in the scientific review of a paper 2. Reviewers should not upload the paper, or any part of it, into an AI tool 3. Reviewers should not upload their peer review comments into an AI tool, even if it is just for the purpose of improving language and readability
Intensive Care Medicine	No description
American Journal of Respiratory and Critical Care Medicine	No description
Critical Care	No description
Chest	No description
Critical Care Medicine	No description
Annals of Intensive Care	No description
Journal of Intensive Care	No description
Resuscitation	No description
Anaesthesia Critical Care and Pain Medicine	No description

## Data Availability

The raw data of the current study are available from the corresponding author on reasonable request.
